# A Computational Approach to Predictive Modeling Using Connection-Based Topological Descriptors: Applications in Coumarin Anti-Cancer Drug Properties

**DOI:** 10.3390/ijms26051827

**Published:** 2025-02-20

**Authors:** Sakander Hayat, Suha Wazzan

**Affiliations:** 1 Faculty of Science, Universiti Brunei Darussalam, Jln Tungku Link, Gadong BE1410, Brunei; 2 Department of Mathematics, Science Faculty, King Abdulaziz University, Jeddah 21589, Saudi Arabia; swazzan@kau.edu.sa

**Keywords:** cheminformatics, mathematical chemistry, topological descriptor, structure–property modeling, physicochemical property, benzenoid hydrocarbon, coumarin compound, 05C92, 05C09

## Abstract

Cheminformatics bridges chemistry, computer science, and information technology to predict chemical behaviors using quantitative structure–property relationships (QSPRs). This study advances QSPR modeling by introducing novel connection-based graphical invariants, specifically designed to enhance the predictive accuracy for physicochemical properties (PCPs) of benzenoid hydrocarbons (BHs). Employing cutting-edge computational methods, we evaluate these invariants against established descriptors in modeling the normal boiling point and standard heat of formation. The findings reveal superior predictive performance by newly proposed invariants, such as the sum-connectivity connection index, outperforming traditional indices like the Zagreb connection indices. Furthermore, we extend these methods to model the physicochemical properties of coumarin-related anti-cancer drugs, demonstrating their potential in drug development. The statistical analysis suggests that the most appropriate structure–property models are nonlinear. This work not only proposes robust tools for PCP estimation but also advocates for rigorous testing of descriptors to ensure relevance in cheminformatics.

## 1. Introduction

### 1.1. Structure–Property Modeling and Topological Descriptors

Quantitative structure–property relationship (QSPR) studies [1] play a vital role in theoretical chemistry, enabling the estimation of thermodynamic and physicochemical properties of molecular structures, and particularly, organic compounds of these predictive models are built upon advanced mathematical and computational methodologies [2]. The origins can be traced back to Harold Wiener [3], who introduced the concept of the “path number”, representing the sum of pairwise distances, to estimate the boiling points of alkanes. This measure was later formalized as the Wiener index in graph theory. Today, QSPR modeling relies on structure-based molecular descriptors [4], which provide the necessary mathematical frameworks. Among these descriptors, topological indices—also referred to as graph-related molecular invariants—have been widely studied [5]. These invariants transform the molecular structure [6], excluding hydrogen atoms, into numerical values that capture essential chemical characteristics. To accurately predict physicochemical properties such as boiling point and heat of formation [7], graphical invariants utilize regression equations [8] that encode the structural characteristics of a compound and its underlying chemical information. For diverse applicability of graph theory and network analysis in other areas such as communication and mobile networks, we refer to [9,10,11,12].

Graphical invariants can be categorized into various types based on the structural properties they incorporate, including degree-related descriptors [13,14], distance-based indices [15], spectral descriptors derived from graph matrices [16], and counting-related polynomials and invariants [17]. With the continuous introduction of new invariants [18], many of them lack substantial chemical relevance [4]. Addressing this issue requires strict criteria for evaluating newly proposed descriptors. Gutman and Tošović [19] highlighted that the proliferation of graphical invariants without rigorous assessment has resulted in an excessive number of descriptors, many of which are unnecessary. This underscores the need to systematically examine emerging families of graphical descriptors for their effectiveness in structure–property modeling, ensuring that only the most efficient ones are advanced while eliminating those that do not contribute meaningfully.

Recent research in mathematical chemistry has increasingly focused on evaluating graphical invariants to determine their effectiveness in predicting physicochemical and thermodynamic properties. This process involves filtering out less reliable descriptors while highlighting those with superior predictive power. The groundwork for this comparative approach was laid by Gutman and Tošović [19], whose study emphasized the importance of systematically assessing molecular descriptors. Their methodology was later extended by Malik et al. [20], who applied it to benzenoid hydrocarbons (BHs), expanding beyond the initial work on isomeric octanes. Additionally, Hayat et al. [21] explored degree-based invariants for their potential to estimate the total π-electronic energy $E_{\pi }$ in BHs. This line of research was further developed in the context of distance-based descriptors [22,23], and subsequent investigations have examined the predictive power of eigenvalue-related graphical invariants [24].

This paper studies a novel family of graphical invariants known as connection-based graphical invariants and conduct a comparative testing to computer their potential to predict PCPs of BHs. Following Gutman and Tošović [19], the standard heat of formation $\Delta_{hf}$ and normal boiling point $\rho_{bp}$ were chosen to represent PCPs. Some new connection-based indices have also been proposed. A computer-dependent computational method is presented to calculate all existing connection-based invariants and contemporary statistical tools such as multiple regression analysis are employed afterwards to rule out insignificant indices while putting forward the ones which deserve further attention in QSPR models. The experimental analysis showcased that the general sum-connectivity connection index outperforms all the connection-based invariants, whereas the well-studied two Zagreb connection indices deliver poor performance. The results in this paper contribute to the avoidance of proliferation of graphical invariants.

### 1.2. Coumarin-Related Compounds

Coumarins are crystalline, colorless polyphenolic compounds classified under oxygenated heterocyclic substances. These compounds were first discovered by Vogel in 1820, who isolated them from the Fabaceae plant Dipteryx odorata Willd., commonly known as “coumaroun” [25]. Oxygenated heterocyclic compounds are a group that includes furan derivatives, containing four carbon atoms, and pyran derivatives, which have five carbon atoms. Furan derivatives are uncommon in plants, but pyran derivatives are more widespread, forming the backbone of numerous compounds like ketones, including α-pyrones and γ-pyrones. When pyran derivatives fuse with benzene in plants, they yield secondary metabolites known as benzo-α-pyrones (coumarins) and benzo-α-pyrones (chromones) [26].

Coumarin (1,2-benzopyrone or 2H-1-benzopyran-2-one) and its derivatives are plant-derived compounds that can be found as glycosides (heterosides) or in their unbound form. To date, nearly 800 naturally occurring coumarin derivatives have been discovered across approximately 600 genera within over 100 plant families [27]. These compounds are commonly found in seeds, roots, and leaves [28], with significant concentrations in plant families like Rutaceae and Apiaceae, which belong to the Dicotyledonae class of the Spermatophyta division. While the majority of natural coumarins are produced by vascular plants, certain types, such as novobiocin, coumermycin, and aflatoxin, are generated by microbial sources [29].

Due to their diverse biological activities, coumarin derivatives have attracted significant attention in recent years. Research has highlighted their potential in various therapeutic areas, including antitumor applications [30], photochemotherapy, anti-HIV activity [31], antibacterial and antifungal properties [32], anti-inflammatory effects [33], and anticoagulant activity through the inhibition of VKOR (vitamin K epoxide reductase) [34]. They also exhibit triglyceride-lowering effects [35] and act as central nervous system stimulants [36]. Hydroxycoumarins, in particular, are noted for their strong antioxidant capabilities, protecting against oxidative stress by neutralizing reactive oxygen species [37]. Furthermore, coumarins with reduced estrogenic activity have been identified, enabling their use in alleviating menopausal symptoms [38]. On the other hand, some coumarin derivatives are employed as flavor enhancers in tobacco products, as noted in [39].

Recently, Timmanaikar et al. [40] employed certain graphical indices in structure–property modeling of coumarin and related compounds. Using several degree-based molecular descriptors, including the Balban index and connective eccentric index (CEI), the study models various physicochemical properties of these compounds, such as boiling point and vapor pressure. Findings reveal that the Balban index and CEI are particularly effective in predicting these properties with high accuracy, highlighting their potential as robust tools in the design of anti-cancer drugs. This work underscores the relevance of topological indices in drug discovery, facilitating efficient and cost-effective molecular analysis. One of the limitations of their work is that their study’s reliance on specific molecular descriptors, like the Balban index and CEI, limits its applicability to broader descriptor classes or additional pharmacological properties.

In this paper, we essentially extended their work to address the aforementioned limitation.

## 2. Results and Discussion

This section delivers a detailed analysis of MLCs in Section 3.3.

The very first unexpected outcome that we observe is that the two multiplicative Zagreb connection descriptors, i.e., Πci(i=1, 2), showcase a considerably poor performance as the MLC for both Πci(i=1, 2) is <0.7, which in structure–property studies is considered very poor. Thus, the study by Javaid et al. [41], who proposed the two multiplicative Zagreb connection invariants, has no meaningful applicative potential from a structure–property studies perspective. We discourage authors from further investigating these two descriptors. Furthermore, the MLC value for the 2nd Zagreb connection invariant Mc2 is <0.9, which from the perspective of mathematical investigation is feasible; however, it does not find popularity among researchers.

Notably, the performance of the 1st Zagreb connection invariant Mc1 with MLC ρ=0.9138 seems reasonable.

Secondly, among the newly proposed connection-based invariants, we observe that the Randić Rc and the general Randić Rcβ connection descriptors with β=1, 2, −2 are less-efficient. However, the general Randić connection descriptor Rc−1 delivers a reasonable performance with ρ=0.9164. The only other newly proposed connection invariant is the augmented Zagreb connection invariant, i.e., AZIc with MLC ρ<0.9. Apart from these connection invariants that deliver poor performance, all other newly proposed connection invariants showcase significantly improved efficiency. For instance, the general sum-connectivity SCcβ connection index with β=−1 records the strongest MLC of ρ=0.9336 and this connection index is among the newly proposed graphical connection-dependent invariants.

The strong potential of the newly proposed connection invariants, such as SCc−1, motivates us to look for other potential connection invariants. Our experiment shows that the general sum-connectivity SCcβ connection index with β=−1, the sum-connectivity connection descriptor SCc, the atom-bond-connectivity ABCc connection index, the geometric-arithmetic GAc connection invariant, and the arithmetic–geometric AGc connection index are among the top five connection-based graphical invariants for estimating the physicochemical characteristics of BHs. It is noteworthy to observe that all of these five best connection descriptors are the newly proposed connection-based invariants, which ultimately justify considering introducing new connection graphical invariants. Table 1 depicts the list of the five best connection-related graphical invariants.

Next, we conduct a detailed statistical analysis of the five best connection-related graphical invariants. First, we put forward appropriate data-fitting multiple linear regression (MLR) models between the PCPs $\rho_{bp}$ and $\Delta_{hf}$ and the five best connection-related graphical invariants. Table 2 delivers the most appropriate data-fitting MLRs with 95% confidence intervals for the intercept and the two *X*-variables. Moreover, Table 2 computes the standard error of estimation and the determination coefficient for the top five connection-related graphical invariants.

We performed the leave-one-out cross validation (LOOCV) method on the data and generated LOOCV root mean squared errors $LOOCV_{RMSE}$ corresponding to the predictions in Table 2. Next, we deliver these cross-validation results.

Table 3 reports $LOOCV_{RMSE}$ for the predictions by the top five connection indices in Table 2. Note that $LOOCV_{RMSE}$ in Table 3 are fairly close to *s* values in Table 2 which shows the efficiency of our predictive models.

**Figure 1:**
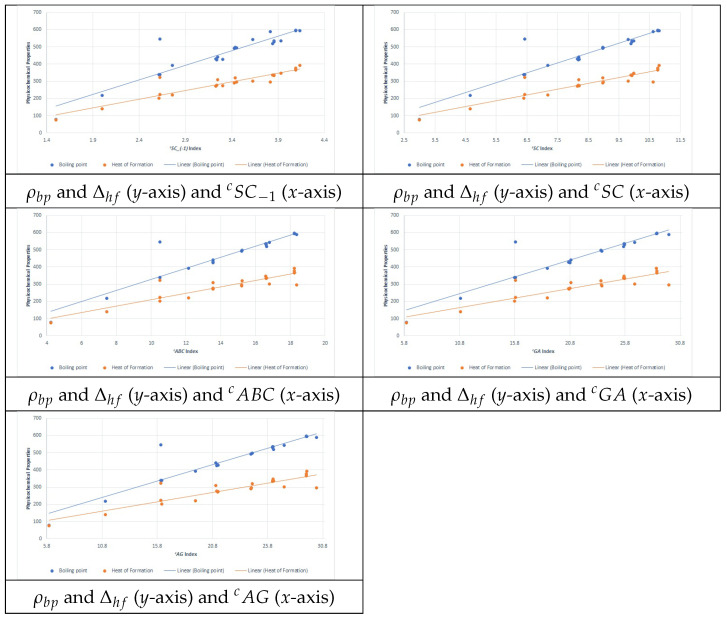
Plot for scattering data between the PCPs $\rho_{bp}$ and $\Delta_{hf}$ and the five best connection-related graphical invariants.

Figure 1 present the plots for scattering data between the PCPs $\rho_{bp}$ and $\Delta_{hf}$ and the five best connection-related graphical invariants.

Note that the general sum-connectivity connection index (SCcβ) has been identified as the best predictor among the tested topological descriptors. This superior predictive power can be attributed to several key theoretical and structural factors:

*Balanced Sensitivity to Molecular Connectivity* 
  Unlike degree-based or distance-based indices, SCcβ incorporates both local and global molecular connectivity by summing the inverse square roots of vertex connections. This ensures a smooth variation across different molecular structures, preventing excessive dependence on extreme values (as seen in multiplicative indices like Πc1 and Πc2).

*Mathematical Robustness and Stability* 
  The formula for SCcβ
  SCcβ=∑ij∈EΩconvi+convjβ
  allows for flexible tuning via the parameter β, enabling it to capture nonlinear structure–property relationships more effectively than rigid indices like the Zagreb connection indices. Empirical results indicate that the β=−1 case provides optimal correlation, likely due to its ability to moderate the influence of high-degree vertices while maintaining significant contributions from lower-degree vertices.

*Strong Correlation with van der Waals Interactions* 
  The sum-connectivity framework aligns well with intramolecular interactions, particularly van der Waals forces and dispersion interactions. These weak but cumulative effects influence key physicochemical properties like boiling points and heat of formation, which were the primary test variables in our study.

*Empirical Evidence from Regression Models* 

- SCcβ achieved the highest multiple correlation coefficient (ρ=0.9336) across all tested indices.
- Regression analysis demonstrated that quadratic and cubic models using SCcβ provided the best predictive accuracy, suggesting that its mathematical form aligns well with nonlinear structure–property relationships.

### 2.1. Structure–Property Modeling of Coumarin-Related Anti-Cancer Drugs

#### 2.1.1. Coumarin-Related Compounds as Potential Anti-Cancer Drugs

Coumarin-related compounds, derived from the benzopyrone family, have garnered significant attention in medicinal chemistry for their diverse biological activities [29], particularly their anti-cancer properties. These compounds exhibit a range of pharmacological actions, including anti-proliferative, pro-apoptotic, and anti-angiogenic effects, which make them promising candidates for cancer therapy [30]. The anti-cancer potential of coumarins is attributed to their ability to modulate various molecular targets, such as inhibiting tyrosine kinases, disrupting cell cycle progression, and inducing oxidative stress in cancer cells. Additionally, coumarins can act as chemosensitizers, enhancing the efficacy of conventional chemotherapy drugs and overcoming drug resistance in certain cancer types.

Coumarins also show potential for selective toxicity, targeting cancer cells while sparing healthy tissues. Their versatility allows for structural modifications, enabling the development of derivatives with enhanced potency and specificity against different cancer types [30]. Examples include esculetin and umbelliferone, which exhibit notable anti-cancer activity through the inhibition of cell signaling pathways and the suppression of metastasis. Furthermore, coumarin derivatives have demonstrated synergistic effects when combined with other anti-cancer agents, highlighting their utility in combination therapy strategies. These compounds represent a promising area of research for developing novel and effective treatments for a wide range of malignancies.

Coumarins exhibit a variety of structural types that contribute to their diverse biological activities [26]. Simple coumarins, the most basic form, consist of a benzopyrone core and are widely found in nature. Furanocoumarins, characterized by a fused furan ring, are known for their photoreactive properties and are often studied for their anti-cancer and anti-inflammatory effects. Pyranocoumarins, with an additional pyran ring, exhibit enhanced lipophilicity and improved bioavailability [28], making them suitable for pharmaceutical applications. Pyrone-substituted coumarins, in which the lactone moiety is modified, demonstrate unique biochemical interactions that broaden their therapeutic potential. These structural variations allow for extensive functional diversity, enabling tailored applications in the design of anti-cancer drugs.

In this paper, we consider 25 contemporary anti-cancer drugs and conduct structure–property modeling of their physicochemical properties. These drugs and their transformed molecular graphs are delivered in Table 4, Table 5 and Table 6. Moreover, Table 4 shows simple coumarin-related compounds, whereas Table 5 (resp. Table 6) records furanocoumarins (resp. pyranocoumarins and pyrone-substituted coumarins) considered in this work. The data of these coumarin compounds were taken from Küpeli et al. [42] who conducted a structure–property study on these compounds. For more on the chemistry of these compounds, we refer to [43,44].

For our structure–property predictive modeling, we consider a diverse range of physicochemical properties, including boiling point (BP) in °C at 760 mmHg, molar volume (MV) in m^3^/mol, enthalpy of vaporization (E) in kJ/mol, density (D) in g/cm^3^, surface tension (ST) cm^3^, vapor pressure (VP) in mmHg at 25 °C, molar refractivity (MR) in A^2^, index of refraction (IR) in cm^3^, flash point (FP) in °C, polarizability (P) in dyne/cm, and polar surface area (PSA) cm^3^. We retrieved the experimental data of these physicochemical properties from the open source http://www.chemspider.com/ (accessed on 15 February 2025). Table 7 delivers the experimental values of these properties for the selected 25 coumarin-related anti-cancer drugs.

#### 2.1.2. Structure–Property Modeling of Physicochemical Properties

Now, we conduct a detailed correlation and regression analysis of the coumarin-related drugs from Table 4, Table 5 and Table 6 with their physicochemical properties in Table 7. We employ the top five connection-based topological descriptors from Table 1 as our predictors.

With three types of regression models, linear, quadratic, and cubic, we evaluated the relationship between molecular descriptors and their hyper-counterparts with 11 essential physicochemical properties of anti-cancer drugs derived from coumarins. The objective of this study was to determine how well these descriptors could predict properties that are crucial to the effectiveness and stability of anti-cancer drugs. In order to evaluate the quality of each model, two key statistical parameters were used: the correlation coefficient (*r*), a measure of the strength and direction of linear relationships between variables, and the standard error of estimate (*s*), a measure of how accurate regression models are. As linear models evolved into cubic models, their complexity and accuracy increased.

The linear regression model provided insight into the relationship between molecular descriptors and drug properties, with molar refractivity (MR) and polarizability (P) showing the highest correlation coefficients. According to Table 8, these properties have a direct and significant linear relationship, suggesting their predictability through linear modeling.

Quadratic regression improves fitting compared to linear models for several properties, such as molar volume (MV) and polarizability (P). According to Table 9, these improvements suggest that the physicochemical properties of these drugs change nonlinearly with changes in molecular descriptors. By including squared terms, the model can capture a wider range of dynamics and variances in data that are not captured by linear models.

As shown in Table 10, the cubic regression model, which incorporates third-degree terms, provides the highest level of precision. It was particularly effective in capturing intricate dependencies on flash point (FP) and molar volume (MV), where higher-order interactions between molecular descriptors are evident. Based on its advanced fit for such properties, the cubic model suggests that some physicochemical traits are influenced by complex interactions that are only captured by higher-order models.

Gradually moving from linear to cubic regressions provides a deeper understanding of drug characteristics. They provide crucial insight into drug efficacy and stability, which is essential for designing optimized anti-cancer drugs. Advances in statistical techniques make it possible to comprehend and predict pharmaceutical behaviors more accurately. This methodology enhances drug development by exploring molecular descriptors and drug properties. It demonstrates the importance of applying advanced statistical tools to pharmaceutical research to improve drug efficacy and patient outcomes by predicting and refining drug properties based on molecular descriptors.

#### 2.1.3. Regression Models for FP, MR, MV, and P

The best cubic regression model is obtained with the geometric-arithmetic connection index:

FP=25.112(GAc)−1.000(GAc)2+0.15(GAc)3−22.874,r=0.926,s=25.971,f=42.250.

This model is based on the arithmetic–geometric connection index:

FP=23.407(GAc)−0.882(GAc)2+0.013(GAc)3−18.121,r=0.926,s=26.029,f=42.028.

**Figure 2:**
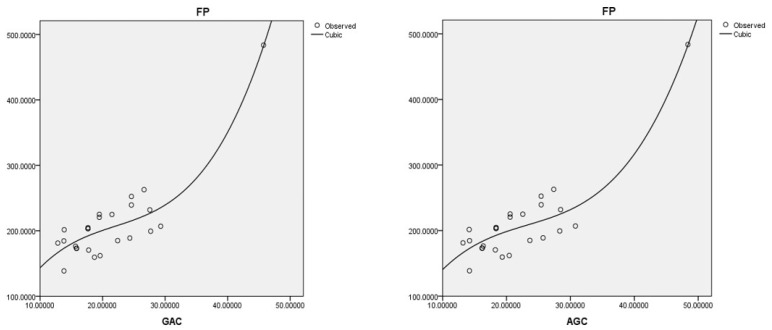
Regression models for FP with the best fit to the data.

Coumarins and their related compounds represent the best overall model for anti-cancer drugs. They are shown in Figure 2.

The best cubic regression model is obtained with the sum-connectivity connection index:

MR=28.662(SCc)−2.105(SCc)2+0.160(SCc)3−12.465,r=0.984,s=4.660,f=208.971.

The best linear and quadratic regression model is obtained with the sum-connectivity connection index:

MR=20.485(SCc)−2.863,r=0.983,s=4.481,f=677.655.

MR=19.069(SCc)+0.156(SCc)2−0.038,r=0.984,s=4.566,f=326.389.

**Figure 3:**
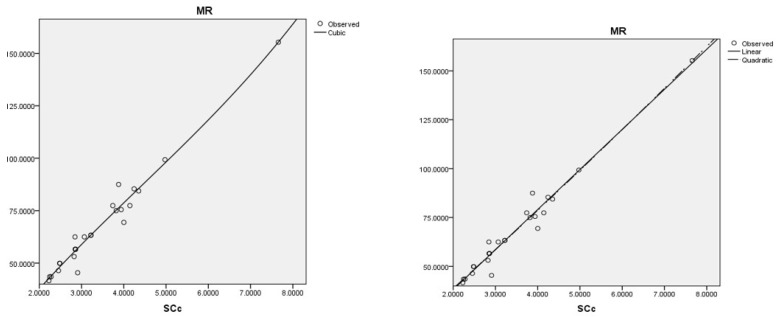
Appropriate regression models for MR with the data.

Coumarins and their related compounds represent the best overall model for anti-cancer drugs, as shown in Figure 3.

The best cubic regression model is obtained with the sum-connectivity connection index:

MV=82.583(SCc)−6.572(SCc)2+0.495(SCc)3−36.530,r=0.954,s=22.237,f=70.461.

The best linear and quadratic regression model is obtained with the sum-connectivity connection index:

MV=56.763(SCc)−5.7903,r=0.954,s=21.299,f=230.309.

MV=52.884(SCc)+0.426(SCc)2+1.945,r=0.954,s=21.753,f=110.422.

Coumarins and their related compounds represent the best overall model for anti-cancer drugs.

**Figure 4:**
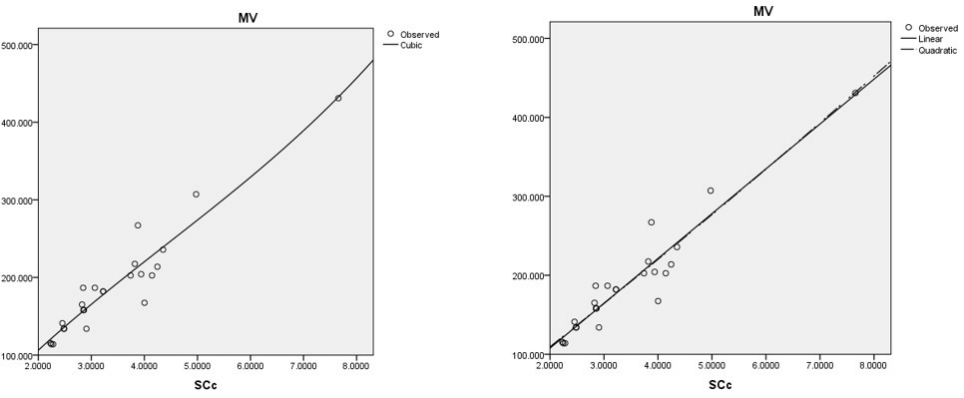
Data-fitting regression models for MV with the data.

They are shown in Figure 4.

The best cubic, linear, and quadratic regression model is obtained with the sum-connectivity connection index, as follows:

P=10.955(SCc)−0.741(SCc)2+0.057(SCc)3−4.390,r=0.984,s=1.852,f=208.023.P=8.124(SCc)−1.133,r=0.983,s=1.781,f=674.950.P=7.536(SCc)+0.065(SCc)2+0.039,r=0.984,s=1.814,f=325.296.

Coumarins and their related compounds represent the best overall model for anti-cancer drugs.

**Figure 5:**
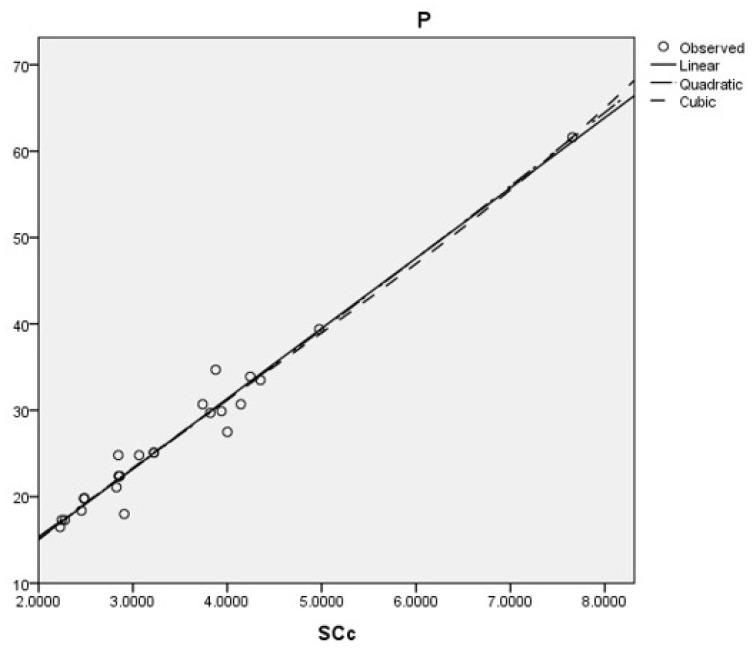
Regression models for *P* that best fit the data.

The regression models are shown in Figure 5.

Although applying these results in drug discovery pipelines demands a separate study, we recall a seminal work by Estrada et al. [45], which addresses this gap of structure–property modeling by graphical descriptors and drug discovery research. The reader is referred to this work for an illustration of the role of graphical descriptors in drug discovery research.

## 3. Materials and Methods

### 3.1. Mathematical Preliminaries

A graph Ω is a pair $\left(V_{\Omega },E_{\Omega }\right)$ in which $V_{\Omega }$ is the vertex set and EΩ⊆VΩ2. The valency/degree $\deg_{x}$ of a vertex $x\in V_{\Omega }$ is defined as $\deg_{x}=|\left\{z\in V_{\Omega }:xz\in E_{\Omega }\right\}|$. The distance/geodesic dis(x,z) between two vertices $x,z\in V_{\Omega }$ has the definition $\mathrm{dis}\left(x,z\right):=\min\{\ell \left(P_{x,z}\right)\}$, where $\ell (P_{x,z})$ is the length (number of edges traversing) by the path $P_{x,z}$ (chain of vertices connecting *x* to *z*). Based on the geodesic, we define the connection ${\mathrm{con}}_{x}$ of vertex $x\in V_{\Omega }$ as ${\mathrm{con}}_{x}:=|\left\{z\in V_{G}:\mathrm{dis}\left(x,z\right)=2\right\}|$, i.e., the number of vertices at distance two from *x*.

A graphical invariant is said to be connection-based if it is structured on the vertices’ connection. The next subsection surveys all the existing connection invariants. Some new connection-based indices have also been put forward.

#### Connection-Based Graphical Invariants

Based on the connection of vertices, the first two connection-based graphical descriptors were proposed by Ali and Trinajstić [46]. They defined the first Zagreb connection index as follows:

(1) Mc1=∑ij∈EΩconvi+convj

They also introduced a degree-connection-based descriptor called the modified first Zagreb connection index. It is defined as

Mdc1=∑i∈VΩdegviconvi

Ali et al. [47] studied Mdc1 of certain T-sum graphs. Ali and Trinajstić [46] also studied the applicability of these descriptors in cheminformatics. Immediately after its conception, Ducoffe et al. [48] derived extremal graphs corresponding to Mc1. Tang et al. [49] in 2019 put forward the second Zagreb connection index as follows:

(2) Mc2=∑ij∈EΩconviconvj

They proved some results corresponding to Mci(i=1,2) for certain derived graphs, such as semi-total point/line graph etc. Cao et al. [50] studied molecular graphs with respect to some operation for Zagreb connection indices. Javaid et al. [41] delivered the multiplicative version of two Zagreb connection invariants. They are defined as follows:

(3) Πc1=∏ij∈EΩconvi+convj

(4) Πc2=∏ij∈EΩconviconvj

Moreover, these multiplicative Zagreb connection invariants were further investigated for wheel-related graphs.

Note that Diudea et al. ([8], Chapter 4) presented a rationale to be followed while proposing a graphical descriptor. It includes a list of the following desirable attributes for constructing a graphical descriptor:

1.  Direct structural interpretation;
2.  Good correlation with at least one property;
3.  Good discrimination of isomers;
4.  Locally defined;
5.  Generalizable to higher analogs;
6.  Linearly independent;
7.  Simplicity;
8.  Not trivially related to other indices;
9.  Efficiency of construction;
10. Based on familiar structural concepts;
11. Show a correct size dependence;
12. Gradual change with gradual change in structures.

We observed that most of the existing connection-based graphical descriptors failed to comply with these aforementioned attributes. For instance, the modified first Zagreb connection index Mdc1 and the two multiplicative Zagreb connection indices Πci(i=1,2) fail to comply with attributes such as numbers 4, 5, and 6. Building upon this limitation, we introduce novel connection graphical indices efficiently complying with the theoretical foundation delivered by Diudea et al. ([8], Chapter 4).

Note that connection-based descriptors are introduced based on their degree-based counterparts; see [46]. Following this, we further enhanced this study by proposing connection descriptors based on other degree-based graphical invariants. Note that the references cited here are regarding the corresponding degree-based graphical invariants.

The following expression delivers the Randić [51] connection index Rc of Ω.

(5) Rc=∑ij∈EΩ1convi×convj.

For β∈R∖{0}, the general Randić [52] connection index Rcβ of Ω has the following mathematical formula:

(6) Rcβ=∑ij∈EΩconvi×convjβ.

Note that Rc−12=Rc.

Next, we put forward the sum-connectivity [53] connection index SCc

(7) SCc=∑ij∈EΩ1convi+convj,

and the general sum-connectivity [54] connection index SCcβ

(8) SCcβ=∑ij∈EΩconvi+convjβ

where β∈R∖{0}. Note that SCc−12=SCc.

The atom-bond connectivity [55] connection index ABCc possesses the defining structure:

(9) ABCc=∑ij∈EΩconvi+convj−2convi×convj.

Next, we introduce the augmented Zagreb [56] connection index AZc as follows:

(10) AZIc=∑ij∈EΩconvi×convjconvi+convj−23.

The geometric–arithmetic [57] and the arithmetic–geometric [58] connection index has the mathematical expression

(11) GAc=∑ij∈EΩ2convi×convjconvi+convj.

and

(12) AGc=∑ij∈EΩconvi+convj2convi×convj.

respectively.

The reduced Randić [59] connection RRc and the reduced reciprocal Randić [60] connection index RRRc are defined as

(13) RRc=∑ij∈EΩconvi×convj.

and

(14) RRRc=∑ij∈EΩ(convi−1)(convj−1).

respectively.

Finally, the Sombor [61] connection index SOc is defined as

(15) SOc=∑ij∈EΩconvi2+convj2.

**Figure 6:**
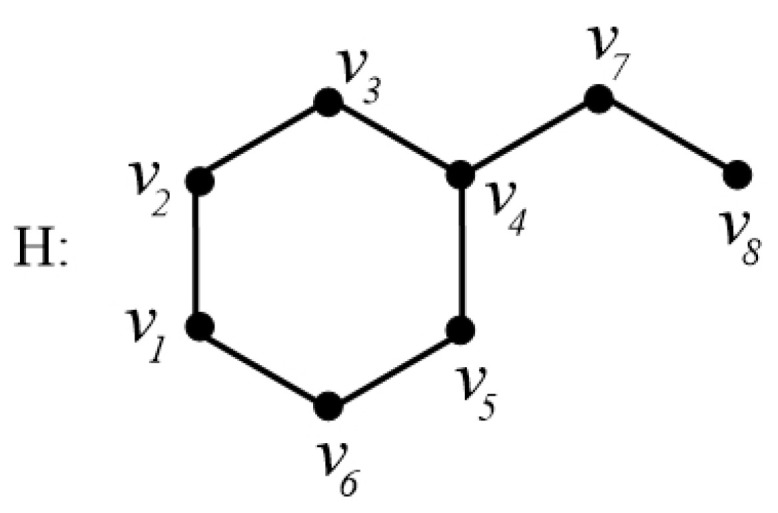
A graph H=(V,E) with the vertex set $V=\{v_{1},v_{2},\ldots ,v_{8}\}$ and edge set $E=\{v_{1}v_{2},v_{2}v_{3},v_{3}v_{4},v_{4}v_{5},v_{5}v_{6},v_{4}v_{7},v_{7}v_{8}\}$.

In order to explain these connection-based indices, we consider the example of a chemical graph *H* in Figure 6.

The graph *H* in Figure 6 has order 8. The connection ${\mathrm{con}}_{v_{4}}$ of the vertex $v_{4}$, for instance, is calculated as

$${\mathrm{con}}_{v_{4}}=\left|\left\{v_{2},v_{6},v_{8}\right\}\right|=3.$$

That is, there are three vertices $v_{2},v_{6},v_{8}$ which are at distance 2 from $v_{4}$. Thus, we have ${\mathrm{con}}_{v_{4}}=3$. In a similar fashion, by calculating the connections of all the vertices of *H*, let us calculate the connection index AZIc as follows:

AZIc(H)=∑ab∈Econa×conbcona+conb−23.

For graph *H* in Figure 6, its AZI index can be calculated as follows:

AZIc(H)=conv1conv2conv1+conv2−23+conv2conv3conv2+conv3−23+…+conv7conv8conv7+conv8−23=2×22+2−23+2×32+3−23+…+1×21+2−23=59.3906.

**Figure 7:**
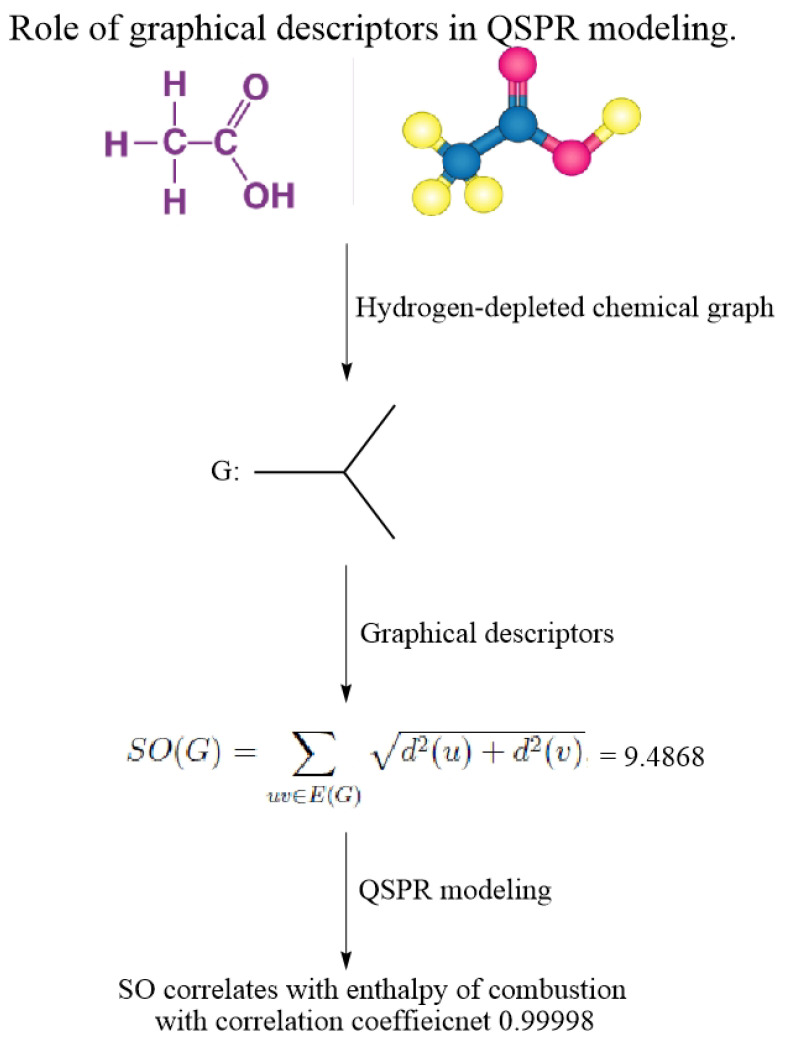
A framework of workflow for this study.

Figure 7 explains the workflow of employing graphical descriptors in structure–property modeling.

Next, we deliver computational details applied in this study.

### 3.2. Computational Methods

This section is dedicated to presenting a computer-dependent computing technique to calculate connection-based graphical indices presented in Section 3.1.

The method makes use of three software packages simultaneously. This includes a computational chemistry software called HyperChem (version 8.0) [62], a mathematical platform to conduct matrix analysis, i.e., MatLab (version R2024b) [63], and TopoCluj (version 1.1) [64], a molecular topology platform. HyperChem is a comprehensive molecular modeling and computational chemistry software widely used in academic and industrial research. Its functionalities span various aspects of molecular modeling, including molecular mechanics, quantum chemistry, molecular dynamics, and visualization. For instance, regarding molecular modeling (resp. quantum chemistry), it performs model building, 3D visualization, etc. (resp. Density Functional Theory (DFT), ab initio methods, etc.). On the other hand, for molecular mechanics (resp. spectroscopy), its ability to conduct energy minimization (resp. UV-Vis and IR spectra, NMR spectroscopy, etc.) possesses significant efficiency. TopoCluj is a specialized software designed for calculating topological descriptors from topological matrices and polynomials. These descriptors are essential in the study of molecular characteristics as well as structures, particularly in the field of computational chemistry and molecular graph theory. MATLAB (short for “Matrix Laboratory”) is an interactive user-friendly environment and a high-level programming language delivered by MathWorks, primarily used for algorithmic development, data analysis, numerical visualization, and numerical computing.

Next, we provide a rationale for selecting the three platforms, i.e., HyperChem, MATLAB, and TopoCluj:

*HyperChem:* HyperChem is a widely used computational chemistry software known for its robust molecular modeling and visualization tools. Unlike other molecular modeling software (such as Gaussian (version 16) or Spartan (version 9)), HyperChem offers the following:

1. User-friendly interface for building and optimizing molecular structures.
2. Real-time visualization of molecular properties and transformations.
3. Efficient quantum mechanics and molecular mechanics calculations, which are particularly useful for cheminformatics applications.

*MATLAB:* MATLAB was chosen for its advanced numerical computing and matrix operations, which are essential for processing topological descriptors. Compared to alternatives like Python 3.13.2 (NumPy, SciPy) or R 4.4.2, MATLAB R2024b offers:

1. Highly optimized built-in matrix operations, crucial for computing large-scale graph-based descriptors.
2. Statistical and regression modeling capabilities, enabling precise correlation analysis.
3. Seamless integration with other scientific tools, ensuring flexibility in extending the analysis.

*TopoCluj:* TopoCluj is a specialized molecular topology software designed for calculating topological indices from molecular graphs. It was preferred over general-purpose tools like ChemOffice or Open Babel due to the following:

1. Dedicated algorithms for computing topological descriptors, reducing computational complexity.
2. Efficient processing of molecular graphs, making it highly suited for cheminformatics applications.
3. Compatibility with standard cheminformatics workflows, ensuring consistency in descriptor computations.

Here, we deliver our proposed 3-step computational method to compute connection-related descriptors for a given molecular graph Ω:

Step 1 Use the HyperChem drawing module to construct a 3D molecular graph of Ω. It delivers a file with the .hin extension.
Step 2 Feed the .hin file to TopoCluj to compute the distance matrix of Ω and generate the .m file corresponding to the .hin file.
Step 3 Compute all connection-related descriptors (from Section 3.1) by inputting .m to MatLab and employing our code written in MatLab.

**Figure 8:**
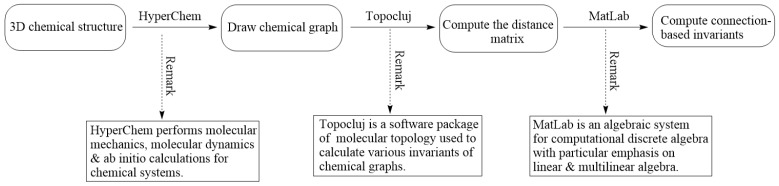
Step-by-step algorithmic steps of the computational method.

Our step-by-step computational method is depicted in Figure 8.

We have made our MatLab code public by employing GitHub platform. Click the GitHub link in order to access all the data.

### 3.3. Data Analysis

This section delivers all the data, their usage, and implications in structure–property modeling.

The first step is to select test properties as representatives of physicochemical properties. Following the seminal work of Gutman and Tošović [19], the normal boiling point $\rho_{bp}$ and standard heat-of-formation $\Delta_{hf}$ were selected as test physicochemical characteristics. The selection of $\rho_{bp}$ is justified, as it represents van der Waals/intramolecular-type reciprocations. Moreover, the justification for opting $\Delta_{hf}$ is that the standard heat-of-formation delivers representation for thermochemical characteristics. Note that $\rho_{bp}$ of a substance is the temperature at which its vapor pressure equals atmospheric pressure (1 atmosphere or 101.3 kPa) at sea level. Each substance has a unique normal boiling point depending on its molecular properties. Moreover, $\Delta_{hf}$ is the change in enthalpy when one mole of a compound is formed from its constituent elements in their standard states under standard conditions (usually 298 K and 1 atm pressure).

Next, as representatives of benzenoid hydrocarbons (BHs), we select 22 lower BHs as our test molecules. This has previously been performed by Hayat and Khan [65] and Hayat et al. [22]. Note that selecting lower BHs for this kind of testing is motivated by Lučić et al. [66], who considered 30 lower BHs for determining the predictive ability of two degree-based graphical descriptors for the total π-electronic energy of BHs. We have considered the lower 22 derivatives because of the limitation of public availability of the experimental data of $\rho_{bp}$ and $\Delta_{hf}$. Hayat and Khan selected lower 22 BHs in their comparative analysis to test the quality of eigenvalue-related degree descriptors. In addition, Hayat et al. [22] opted for 22 lower BHs to investigate the prediction ability of distance-related graph-theoretic invariants for physicochemical characteristics of BHs. The close correlation values of both $\rho_{bp}$ and $\Delta_{hf}$ for the initial 22 members of BHs strengthen the justification for their selection in this study. Moreover, we find the number 22 sufficient in order to validate our statistical inferences. The experimental data of $\rho_{bp}$ and $\Delta_{hf}$ for some BHs were retrieved from NIST’s standard data repository [67], while we consulted Allison and Burgess [7], Dias [68], and Nikolić et al. [69] for the remaining BHs.

**Figure 9:**
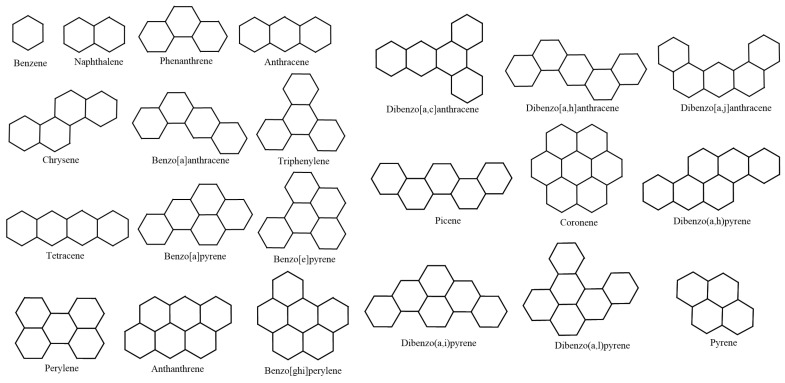
The 2D graphs of the 22 initial members of BHs selected as test molecules.

Figure 9 showcased the graphical structures of the 22 initial members of BHs selected as test molecules. Since these are just the graphical representations of the 3D molecular structure of BHs, the aromaticity is omitted. The next step is to compute numerical values of the connection-related graphical invariants in Section 3.1. In order to do that, the computational method explained in Section 3.2 has been employed. Moreover, Table 11 delivers experimental values of both $\rho_{bp}$ and $\Delta_{hf}$ for lower 22 BHs. The implementation of the proposed method in Section 3.2 for the first/second Zagreb and multiplicative Zagreb connection invariants delivers the data in Columns 4–7. Although we omit the data for the other connection invariants, it is noteworthy to say that those indices can be computed similarly.

The final step of this section is to employ the multiple linear correlation (MLC) between a given connection index $C_{I}$ and the two chosen PCPs, i.e., $\rho_{bp}$ and $\Delta_{hf}$. We compute $\rho :=\rho (\rho_{bp},\Delta_{hf};C_{I})$, i.e., the multiple linear correlation between $X_{1}=\rho_{bp}$, $X_{2}=\Delta_{hf}$, and a connection invariant $Y=C_{I}$ by using the data in Table 11. The data analysis toolpack of MS Excel is utilized for this computation. Table 12 delivers the data of MLC values. Note that the general sum-connectivity SCcβ and Randić Rcβ connection indices has a generic parameter β∈R∖{0}. Thus, for a meaningful analysis, we select β∈{±1,±2} as test values of β∈R∖{0}.

In the next section, we deliver a detailed analysis of the data in Table 12 and mention the top five best connection-based graphical invariants for predicting PCPs of BHs, meanwhile mentioning the ones that do not deserve further attention from the researchers.

## 4. Conclusions

### 4.1. Contributions

1. Introduced novel connection-based graphical invariants for predicting physicochemical properties (PCPs).
2. Demonstrated superior performance of the general sum-connectivity index over traditional indices.
3. Applied models to benzenoid hydrocarbons (BHs) and coumarin-related anti-cancer drugs, advancing QSPR modeling methods.

### 4.2. Study Implications

1. Enhanced predictive accuracy for boiling points, heat-of-formation, and key PCPs.
2. Offered scalable tools for drug development and molecular screening.
3. Provided a foundation for cheminformatics applications in material science and pharmaceuticals.

### 4.3. Limitations

1. Descriptor Selection Bias: The focus on connection-based descriptors may underrepresent quantum chemical and steric effects, requiring hybrid models for broader applicability.
2. Dataset Constraints: This study is limited to benzenoid hydrocarbons and coumarins, restricting generalizability to other chemical classes.
3. Modeling Assumptions: Standard environmental conditions and smooth regression models may not fully capture real-world molecular behavior.
4. Potential Overfitting: The small dataset may lead to overfitting in statistical models, necessitating regularization techniques.

### 4.4. Future Study

1. Expand datasets to include diverse molecular structures.
2. Explore additional properties like toxicity, solubility, and pharmacokinetics.
3. Integrate machine learning for enhanced predictive performance.
4. Investigate real-world applications in drug discovery and advanced material design.

## Figures and Tables

**Table 1:** The top five connection-related graphical invariants.

Position	Connection-Based Index	MLC Value
1	SCc−1, Equation (8) with β=−1	0.9336
2	SCc, Equation (7)	0.9318
3	ABCc, Equation (9)	0.9310
4	GAc, Equation (11)	0.9279
5	AGc, Equation (12)	0.9272

**Table 2:** The most appropriate data-fitting MLR models for the top 5 connection-related graphical invariants and the chosen PCPs $\rho_{bp}$ and $\Delta_{hf}$.

Connection Index	MLR Model	Statistics
SCc−1	SCc−1=0.8551±0.4702+0.0018±0.0033ρbp+0.0018±0.0094Δhf	$r^{2}=0.8716,\;s=0.2644$
SCc	SCc=1.3475±1.4110+0.0105±0.0098ρbp+0.0079±0.0166Δhf	$r^{2}=0.8683,\;s=0.7935$
ABCc	ABCc=1.5566±2.5411+0.0223±0.0177ρbp+0.0081±0.0299Δhf	$r^{2}=0.8667,\;s=1.4290$
GAc	GAc=1.4568±4.2730+0.0416±0.0297ρbp+0.0044±0.0503Δhf	$r^{2}=0.8610,\;s=2.4029$
AGc	AGc=1.4305±4.4032+0.0425±0.0306ρbp+0.0047±0.0519Δhf	$r^{2}=0.8597,\;s=2.4761$

**Table 3:** The leave-one-out cross validation (LOOCV) root mean squared errors for the top five connection indices.

Descriptor	${\mathrm{LOOCV}}_{\mathrm{RMSE}}$
SCc−1	0.2800
SCc	0.84530
ABCc	1.5162
GAc	2.5931
AGc	2.6490

**Table 4:** Chemical structures and their corresponding molecular graphs for certain simple coumarins.

Cancer Drug	Molecular Graph	Cancer Drug	Molecular Graph
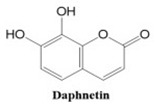	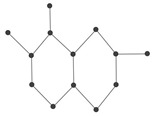	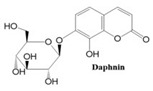	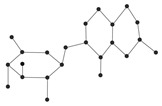
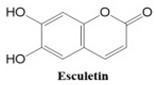	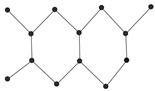	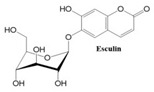	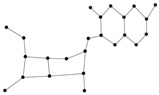
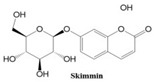	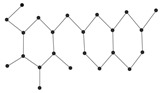	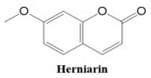	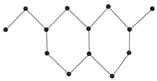
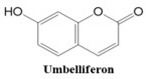	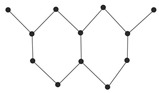	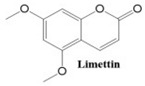	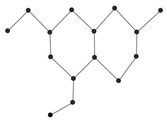

**Table 5:** Some furanocoumarins and their transformed molecular graphs.

Cancer Drug	Molecular Graph	Cancer Drug	Molecular Graph
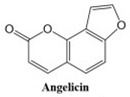	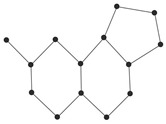	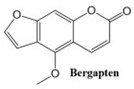	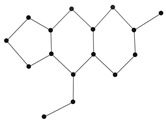
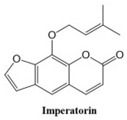	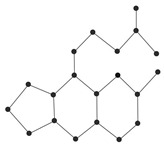	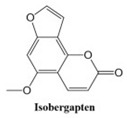	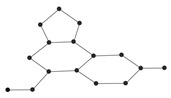
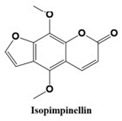	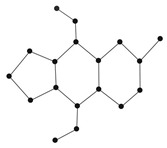	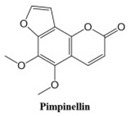	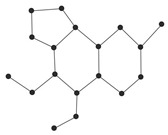
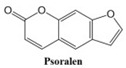	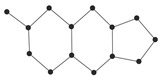	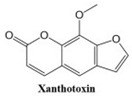	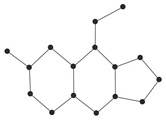

**Table 6:** Chemical structures of some pyranocoumarins and pyrone-substituted coumarins.

Cancer Drug	Molecular Graph	Cancer Drug	Molecular Graph
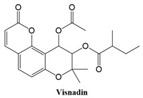	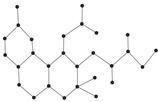	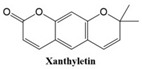	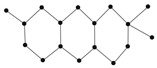
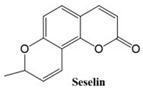	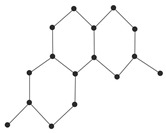	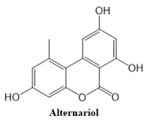	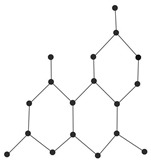
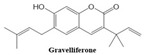	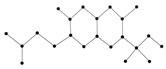	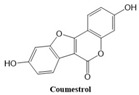	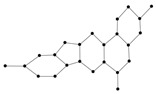
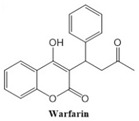	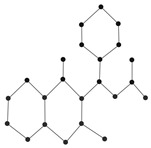	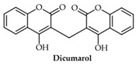	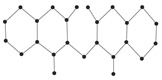
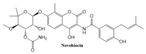	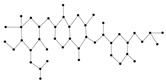		

**Table 7:** The physicochemical properties of the selected coumarin-related anti-cancer drugs.

Drugs	BP	MV	E	D	ST	VP	MR	IR	FP	P	PSA
Alternariol	384.6	186.7	63.3	1.2	42.7	0.9	62.5	1.584	161.9	24.8	36
Angelicin	362.6	134.0	60.9	1.4	55.5	0.8	49.9	1.667	173.1	19.8	39
Bergapten	412.4	158.0	66.5	1.4	52.0	1.0	56.6	1.635	203.2	22.4	49
Coumestrol	406.0	167.4	68.3	1.6	79.9	1.0	69.4	1.768	199.3	27.5	80
Daphnetin	430.4	114.0	71.2	1.6	75.6	1.1	43.5	1.689	184.5	17.3	67
Daphnin	670.0	202.6	103.4	1.7	92.6	2.2	77.4	1.689	252.4	30.7	146
Dicumarol	620.7	213.8	96.7	1.6	80.7	1.9	85.4	1.731	231.9	33.9	93
Esculetin	469.7	114.0	76.0	1.6	75.6	1.2	43.5	1.689	201.5	17.3	67
Esculin	697.7	202.6	107.3	1.7	92.6	2.3	77.4	1.689	262.8	30.7	146
Gravelliferone	454.3	267.1	74.1	1.1	43.3	1.2	87.5	1.569	184.9	34.7	47
Herniarin	335.3	141.1	57.8	1.2	44.4	0.7	46.4	1.572	138.6	18.4	36
Imperatorin	448.3	217.5	70.7	1.2	46.7	1.1	75.0	1.606	224.9	29.7	49
Isobergapten	412.4	158.0	66.5	1.4	52.0	1.0	56.6	1.635	203.2	22.4	49
Isopimpinellin	448.7	182.0	70.7	1.4	49.6	1.1	63.3	1.612	225.1	25.1	58
Limettin	388.1	165.1	63.7	1.2	43.0	0.9	53.1	1.557	176.3	21.1	45
Novobiocin	876.2	431.0	133.4	1.4	70.6	0.0	155.3	1.640	483.7	61.6	196
Pimpinellin	441.0	182.0	69.8	1.4	49.6	1.1	63.3	1.612	220.5	25.1	58
Psoralen	362.6	134.0	60.9	1.4	55.5	0.8	49.9	1.667	173.1	19.8	39
Seselin	403.0	186.7	65.4	1.2	42.7	0.9	62.5	1.584	170.5	24.8	36
Skimmin	632.0	204.2	98.2	1.6	81.4	1.9	75.5	1.661	239.3	29.9	126
Umbelliferon	382.1	115.5	65.5	1.4	59.5	0.9	41.6	1.640	181.2	16.5	47
Visnadin	477.7	307.2	74.2	1.3	49.5	1.2	99.3	1.560	206.9	39.4	88
Warfarin	515.2	235.8	82.9	1.3	58.7	1.4	84.4	1.635	188.8	33.5	64
Xanthotoxin	414.8	158.0	66.8	1.4	52.0	1.0	56.6	1.635	204.7	22.4	49
Xanthyletin	340.0	133.9	58.4	1.3	46.4	0.7	45.4	1.593	159.5	18.0	29

**Table 8:** The $R^{2}$ values for the linear regression model.

Descriptor	D	BP	VP	E	FP	IR	MR	PSA	P	ST	MV
SCc−1	0.004	0.648	0.000	0.638	0.707	0.003	0.967	0.594	0.967	0.079	0.909
SCc	0.016	0.670	0.000	0.662	0.712	0.014	0.950	0.631	0.950	0.112	0.870
ABCc	0.024	0.678	0.001	0.671	0.711	0.022	0.937	0.646	0.937	0.129	0.847
GAc	0.033	0.674	0.002	0.666	0.703	0.032	0.919	0.652	0.919	0.142	0.821
AGc	0.026	0.668	0.001	0.660	0.709	0.024	0.931	0.640	0.931	0.127	0.840

**Table 9:** The $R^{2}$ values for the quadratic regression model.

Descriptor	D	BP	VP	E	FP	IR	MR	PSA	P	ST	MV
SCc−1	0.008	0.649	0.508	0.641	0.826	0.005	0.967	0.599	0.967	0.086	0.909
SCc	0.024	0.672	0.513	0.667	0.840	0.020	0.951	0.637	0.951	0.123	0.872
ABCc	0.035	0.681	0.514	0.677	0.844	0.030	0.939	0.652	0.939	0.142	0.850
GAc	0.043	0.678	0.508	0.675	0.843	0.040	0.924	0.660	0.924	0.154	0.827
AGc	0.036	0.673	0.503	0.668	0.843	0.031	0.935	0.648	0.935	0.137	0.844

**Table 10:** The $R^{2}$ values for the cubic regression model.

Descriptor	D	BP	VP	E	FP	IR	MR	PSA	P	ST	MV
SCc−1	0.050	0.649	0.537	0.642	0.849	0.035	0.968	0.604	0.967	0.151	0.910
SCc	0.134	0.675	0.572	0.672	0.854	0.108	0.952	0.661	0.952	0.274	0.875
ABCc	0.172	0.686	0.588	0.686	0.857	0.143	0.941	0.686	0.941	0.329	0.856
GAc	0.209	0.682	0.589	0.682	0.858	0.184	0.928	0.696	0.928	0.368	0.840
AGc	0.175	0.677	0.579	0.675	0.857	0.151	0.936	0.681	0.936	0.328	0.851

**Table 11:** Experimental values of both $\rho_{bp}$ and $\Delta_{hf}$ for lower 22 BHs and their first/second Zagreb and multiplicative Zagreb connection indices.

Molecule	$\rho_{\mathrm{bp}}$	$\Delta_{\mathrm{hf}}$	Mc1	Mc2	Πc1	Πc2
Benzene	80.1	75.2	24	24	4096	4096
Naphthalene	218	141	64	96	192,080,000	6,879,707,136
Phenanthrene	338	202.7	106	184	7.46807$\times 10^{12}$	8.70713$\times 10^{15}$
Anthracene	340	222.6	104	176	6.29408$\times 10^{12}$	7.2139$\times 10^{15}$
Chrysene	431	271.1	148	273	2.86774$\times 10^{17}$	1.102$\times 10^{22}$
Benzo[a]anthracene	425	277.1	146	265	2.4089$\times 10^{17}$	9.13009$\times 10^{21}$
Triphenylene	429	275.1	150	288	2.62144$\times 10^{17}$	8.30377$\times 10^{21}$
Tetracene	440	310.5	144	256	2.06244$\times 10^{17}$	7.56432$\times 10^{21}$
Benzo[a]pyrene	496	296	184	376	5.35488$\times 10^{20}$	5.14147$\times 10^{26}$
Benzo[e]pyrene	493	289.9	182	363	5.15237$\times 10^{20}$	5.39122$\times 10^{26}$
Perylene	497	319.2	184	374	5.62449$\times 10^{20}$	5.14147$\times 10^{26}$
Anthanthrene	547	323	104	176	6.29408$\times 10^{12}$	7.2139$\times 10^{15}$
Benzo[ghi]perylene	542	301.2	218	466	1.04142$\times 10^{24}$	3.18346$\times 10^{31}$
Dibenzo[a,c]anthracene	535	348	190	370	8.3236$\times 10^{21}$	8.70713$\times 10^{27}$
Dibenzo[a,h]anthracene	535	335	188	354	9.21947$\times 10^{21}$	1.15553$\times 10^{28}$
Dibenzo[a,j]anthracene	531	336.3	188	354	9.21947$\times 10^{21}$	1.15553$\times 10^{28}$
Picene	519	336.9	190	362	1.10121$\times 10^{22}$	1.39471$\times 10^{28}$
Coronene	590	296.7	252	558	1.92829$\times 10^{27}$	1.97112$\times 10^{36}$
Dibenzo(a,h)pyrene	596	375.6	224	456	1.70556$\times 10^{25}$	5.39122$\times 10^{32}$
Dibenzo(a,i)pyrene	594	366	224	456	1.70556$\times 10^{25}$	5.39122$\times 10^{32}$
Dibenzo(a,l)pyrene	595	393.3	226	473	1.62857$\times 10^{25}$	4.54885$\times 10^{32}$
Pyrene	393	221.3	140	270	1.5565$\times 10^{16}$	5.39122$\times 10^{20}$

**Table 12:** MLCs of connection-related graphical invariants with $\rho =\rho (\rho_{bp},\Delta_{hf};C_{I})$ with both $\rho_{bp}$ and $\Delta_{hf}$ for lower 22 BHs.

Connection-Based Index	$\rho =\rho (\rho_{\mathrm{bp}},\Delta_{\mathrm{hf}};C_{I})$
Mc1, Equation (1)	0.9138
Mc2, Equation (2)	0.8938
Πc1, Equation (3)	0.6783
Πc2, Equation (4)	0.6793
Rc, Equation (5)	0.7947
Rc1, Equation (6) with β=1	0.8938
Rc−1, Equation (6) with β=−1	0.9164
Rc2, Equation (6) with β=2	0.8412
Rc−2, Equation (6) with β=−2	0.7950
SCc, Equation (7)	0.9318
SCc1, Equation (8) with β=1	0.9138
SCc−1, Equation (8) with β=−1	0.9336
SCc2, Equation (8) with β=2	0.8927
SCc−2, Equation (8) with β=−2	0.9140
ABCc, Equation (9)	0.9310
AZIc, Equation (10)	0.8892
GAc, Equation (11)	0.9279
AGc, Equation (12)	0.9272
RRc, Equation (13)	0.9143
RRRc, Equation (14)	0.9097
SOc, Equation (15)	0.9132

## Data Availability

Datasets generated or analyzed during the current study are publicly available at https://github.com/Sakander/Connection-based-invariants (accessed on 15 February 2025).
